# Insulin Detemir Causes Lesser Weight Gain in Comparison to Insulin Glargine: Role on Hypothalamic NPY and Galanin

**DOI:** 10.1155/2014/458104

**Published:** 2014-08-14

**Authors:** Mohammad Ishraq Zafar, Cuining Hu, Danfeng Liu, Raja Adeel Shafqat, Feng Gao

**Affiliations:** ^1^Department of Endocrinology, Union Hospital, Tongji Medical College, Huazhong University of Science & Technology, 1277 Jiefang Avenue, Wuhan 430022, China; ^2^Department of Medicine, Tongji Hospital, Tongji Medical College, Huazhong University of Science & Technology, 1095 Jiefang Avenue, Wuhan 430030, China

## Abstract

*Objective.* Compared with other insulin analogues, insulin detemir induces less weight gain. This study investigated whether this effect was achieved by influencing the hypothalamic appetite regulators neuropeptide Y (NPY) and galanin (GAL). *Methods.* Type  2 diabetic rat models were established with a high-fat diet and intraperitoneal injection of STZ. All rats were divided into NC, DM, DM+DE and DM+GLA groups. Glycemic levels of all study groups were checked at study onset and after 4 weeks of insulin treatment. Food intake and body weight were monitored during treatment. After 4 weeks, the hypothalamus of rats was examined for NPY and GAL mRNA and protein expression. *Results.* After 4 weeks of treatment, compared with the DM+GLA group, the DM+DE group exhibited less food intake (*P* < 0.05) and less weight gain (*P* < 0.05), but showed similar glycemic control. The expression of hypothalamic NPY and GAL at both mRNA and protein level were significantly lower (*P* < 0.05) in the DM+DE group. *Conclusion*. Insulin detemir decreased food intake in type 2 diabetic rats, which led to reduced weight gain when compared to insulin glargine treatment. This effect is likely due to downregulation of hypothalamic NPY and GAL.

## 1. Introduction

Diabetes is a common condition that affects a significant percentage of the global population. Unfortunately, its prevalence on a global scale is increasing with time. According to current statistics, 171 million diabetes patients were identified in the year 2000, and it is predicated that 366 million people will be affected by the disease by the year 2030 [1]. Recent results from a 2010 cross-sectional survey based on 98,658 Chinese adults demonstrated that 11.6% were diabetics and 50.1% were prediabetics [2].

Diabetes treatment is often challenging because it involves difficulty in selecting a suitable treatment regimen, determining a patient's glycemic target, frequent blood glucose monitoring, patient compliance, and avoidance of adverse side effects. Combinatorial therapy using insulin with other treatment modalities or insulin use alone is common treatments used for diabetes. However, recombinant insulin use is often accompanied by increased hypoglycemic risk and weight gain [3].

Insulin detemir and insulin glargine are long-acting insulin analogues that are equally efficient in glycemic control. These analogues are identical in their ability to minimize a hypoglycemic event [4, 5], although there are discrepancies in their indication, dosing, and, most importantly, their influence on increasing weight gain. Insulin detemir is preferred over insulin glargine for diabetes treatment during pregnancy. It is considered the safest form of recombinant insulin and has not been implicated in compromising maternal or fetal safety [6]. However, glargine usage is condition dependent, and this analogue is only used if maternal metabolic control is inefficient or the patient has severe diabetic complications [7, 8].

A 52-week treat-to-target comparison trial based on the detemir and glargine insulin analogues demonstrated that patients who received a single dose of insulin detemir had a weight gain of 2.3 kg, whereas those treated with a twice-daily dose of insulin detemir gained 3.7 kg. The weight gain with twice-daily detemir was comparable to the weight gain observed in patients using a single daily dose of glargine [9]. Recent study results have shown that detemir and glargine can be dispensed on a daily basis per the individual needs of patients [10]. It has not yet been determined why detemir promotes less weight gain when compared to insulin glargine in diabetic patients treated with the analogues. There are several hypotheses, but the exact mechanism has not been elucidated thus far [11–13].

The role of neuropeptide Y (NPY) and galanin (GAL) in reducing weight gain in patients treated with insulin detemir versus other insulin analogues is currently unknown. We hypothesized that NPY and GAL are mechanistic targets of detemir and may promote less weight gain in diabetic patients treated with detemir. NPY and GAL are comprised of 36 and 29 amino acids, respectively. They are widely distributed across the peripheral and central nervous systems. Intracerebroventricular (ICV) administration of NPY and GAL can induce food intake in satiated rats [14], and NPY and GAL regulate food intake via the subtype receptors neuropeptide Y receptor type 5 (Y5) and galanin receptor 1 (GAL1), respectively. Selective Y5 and GAL1 antagonists may be effective in treatment of obesity and eating disorders [15–17]. This hypothesis alludes to a potential role for Y5 and GAL1 in food and energy regulation.

The mechanisms of action of NPY and GAL are also affected by additional factors. It has been reported that ICV injection of leptin (4 *μ*g) in rats blocked their urge for food intake, a result that is also observed following ICV administration of GAL or NPY. This study suggests that leptin signals in the hypothalamus may alter the postsynaptic actions of GAL and NPY, leading to decreased food intake [14]. It is unknown whether insulin detemir or glargine will modulate the same or different mechanisms of action on NPY or GAL in the hypothalamus. We hypothesized that the contrasting effects of detemir and glargine on the NPY and GAL mechanisms of action would result in differential weight gain in animals treated with either analogue. Therefore, we conducted a comparative analysis of detemir and glargine using a type 2 diabetes Sprague Dawley (SD) rat model. We investigated the impact of basal insulin on body weight, food intake, and hypothalamic NPY and GAL mRNA and protein expression.

## 2. Materials and Methods

### 2.1. Animal Grouping and Dietary Plan

SD rats (80 to 120 g) were fed a normal diet with adequate hydration and were kept in individual cages under an optimum temperature of 18–25°C with 40–60% humidity. After one week, all rats were weighed and randomly divided into four groups: normal control group (NC); diabetic control group (DM); diabetes plus detemir group (DM + DE); and diabetes plus glargine group (DM + GLA). The high-fat diet (HFD) consisted of 58% fat, 25.6% carbohydrate, and 16.4% protein [18] and was provided to all groups except the NC group for 6 weeks.

### 2.2. Streptozotocin-Induced Diabetes Model and Intervention

Following 6 weeks of feeding the type 2 diabetic SD rats with the defined dietary HFD protocol, all rats (except those in the NC group) were administered intraperitoneal injection of streptozotocin (STZ; 35 mg/kg) according to their fasting weight. Tail venous blood was drawn and measured for glycemic level using the glucose oxidase method. Blood glucose > 16.7 mmol/L was the set criterion for the DM group [19]. The detemir and glargine insulin intervention groups were injected subcutaneously (3 U/day/rat) at study onset, and the dose was later adjusted according to their glycemic level. Rats in the DM + DE and the DM + GLA groups received insulin at a fixed time (10 AM) daily for 4 weeks. The control groups were injected subcutaneously with saline at the same time and for the same duration. Glycemic level, body weight, and food intake were monitored for the rats in each group.

### 2.3. Hypothalamus Excision

After 4 weeks of insulin intervention, all rats were fasted overnight (12 hours) and were sacrificed under anesthesia (3% sodium pentobarbital; 50 mg/kg). The mice were decapitated, and brain tissue was obtained and placed on ice. The midpoint between the optic chiasm and the tuber cinereum was excised to isolate the hypothalamus. The hypothalamus was excised and preserved in tagged storage tubes and submerged in liquid nitrogen. The samples were used to determine mRNA and protein expression for NPY and GAL.

All experimental procedures were performed in a laboratory at Tongji Medical College, Huazhong University of Science & Technology.

## 3. Detection Process

### 3.1. Reverse-Transcriptase Polymerase Chain Reaction (RT-PCR) Detection of NPY and GAL mRNA Expression

Total RNA was extracted from the hypothalamic tissue using TRIzol reagent and the concentration and purity of RNA were measured. The cDNA first chain was synthesized by reverse transcription (Fermentas Kit) and the product was PCR-amplified. The sequences for primers used to detect *β*-actin were 5′-TTCCAGCCTTTCCTGG-3′ (forward) and 5′- TTGCGCTCAGGAGGAGCAAT-3′ (reverse). The reaction conditions used to produce a 226 base pair (bp) product were as follows: 94°C initial denaturation for 3 minutes; 94°C for 30 seconds; 50°C for 30 seconds; 72°C for 30 seconds; 25 cycles; and 72°C terminal extension for 7 minutes.

The sequences for primers used to detect neuropeptide Y (NPY) included 5′-TGGACTGACCCTCGCTCTA-3′ (forward) and 5′-GGGACAGGCAGACTGGTT-3′ (reverse). The reaction conditions used to produce a 348 bp product were as follows: 94°C initial denaturation for 3 minutes; 94°C for 30 seconds; 55°C for 30 seconds; 72°C for 30 seconds; 25 cycles; 72°C terminal extension for 7 minutes. *β*-actin and NPY primers were provided by Shanghai Yingjun Biotechnology Co., Ltd.

The primer sequences used to detect galanin (GAL) were as follows: 5′-GCTCGGGATGCCAACAAAGGA-3′ (forward) and 5′-TGCGGACGATATTGCTCTCAGG-3′ (reverse). The reaction conditions used to produce a 200 bp product were as follows: 98°C for 10 seconds; 53°C for 30 seconds; 72°C for 30 seconds; 30 cycles; and 72°C terminal extension for 7 minutes. The GAL primer was provided by Shanghai Shenggong Biological Engineering Co., Ltd. Agarose RNA gel electrophoresis and gel imaging analysis (UVP Company) were used to analyze the PCR products.

### 3.2. Western Blot Detection of NPY and GAL Protein Expression

The hypothalamic tissue was lysed in RIPA lysis buffer and PMSF to extract all protein. The BCA method was used to determine the concentration of protein. The samples were divided and 5 × loading buffer was added (5 × loading buffer = 4 : 1) and the samples were mixed. The protein mixture was placed in the PCR machine at 95°C for 10 minutes and stored at −20°C.

For Western blot analysis, 40 *μ*g of protein (including 40 *μ*L of protein sample and 5 *μ*L of molecular weight ladder) per sample was resolved on a SDS-PAGE gel (15% separating gel and 5% stacking gel). Gel electrophoresis was performed using 100 v for 1.5 hours with a constant 200 mA current. The gel was transferred to film for 50 minutes, which was then blocked for 1.5 hours. Primary antibodies used for Western blotting were as follows: a mouse anti-rat monoclonal antibody was used to detect NPY (Santa Cruz Company); GAPDH was detected using a rabbit GAPDH anti-rat monoclonal antibody (Wuhan Boster Biotechnology Co., Ltd); a rabbit-derived galanin polyclonal anti-rat antibody (H80) (Santa Cruz Company) was used to detect galanin; and a mouse *β*-actin anti-rat monoclonal antibody (Wuhan Guge Biotechnology Co., Ltd) was used to detect *β*-actin. The membranes were incubated in primary antibody overnight at 4°C and were then rinsed in TBST. Secondary antibodies used in the study were as follows: HRP-labeled goat anti-mouse IgG and HRP-labeled goat anti-rabbit IgG (Wuhan Boster Company) and HRP-labeled goat anti-rabbit IgG and HRP-labeled rabbit anti-mouse IgG (Wuhan Guge Biotechnology Co., Ltd). The membranes were incubated in secondary antibody for 1.5 hours at 37°C, rinsed in TBST, and developed, and the Western blot results were scanned and saved as a digital image.

### 3.3. Data Processing and Statistical Analysis

BIO-RAD Quantity One software was used to quantify each target gene or protein analyzed by RT-PCR or Western blot analysis, respectively. The gray value stripes of each result on digital images were measured using the software. Total mRNA for each gene was normalized using *β*-actin as a control (gray value of each protein stripe/gray value of *β*-actin or GAPDH stripe determined expression of the target genes). GAPDH and *β*-actin were used as controls to normalize data for Western blot analysis. The data were expressed in the form $\overset{-}{x}\pm s$. The statistical software program SPSS16.0 was used to test the homogeneity of variance test. The Bonferroni correction test was performed in cases where the variance was homogeneous. *P* < 0.05 was denoted as significant and *P* < 0.01 was denoted as very significant.

## 4. Results

### 4.1. Food Intake and Weight Measurement

During the 4-week insulin intervention, food intake was recorded for each group (Table 1). Food intake was higher in the diabetic control (DM) group when compared to the diabetes plus detemir (DM + DE) group (*P* < 0.01) and the diabetes plus glargine (DM + GLA) group (*P* < 0.05). The DM + DE group consumed more food than the normal control (NC) group.

Table 2 shows the final weight of rats in each group. The weight of all diabetic rats was significantly lower than the weight of the NC group (*P* < 0.001). The insulin intervention groups demonstrated a higher final weight when compared to the DM group (*P* < 0.05). The DM + DE group exhibited less weight gain than the DM + GLA group (*P* = 0.046) (*P* < 0.05). Overall, results shown here demonstrate that food intake and weight gain corresponded for each group.

### 4.2. Glycemic Status

Following 4 weeks of insulin treatment, tail venous blood was collected from all rats and mean blood glucose was analyzed. The results show that glycemic control by both insulin detemir and insulin glargine was similar with negligible difference (*P* > 0.05) (Table 3).

### 4.3. The Influence of Insulin on Hypothalamic Neuropeptide Y Expression

**Figure 1:**
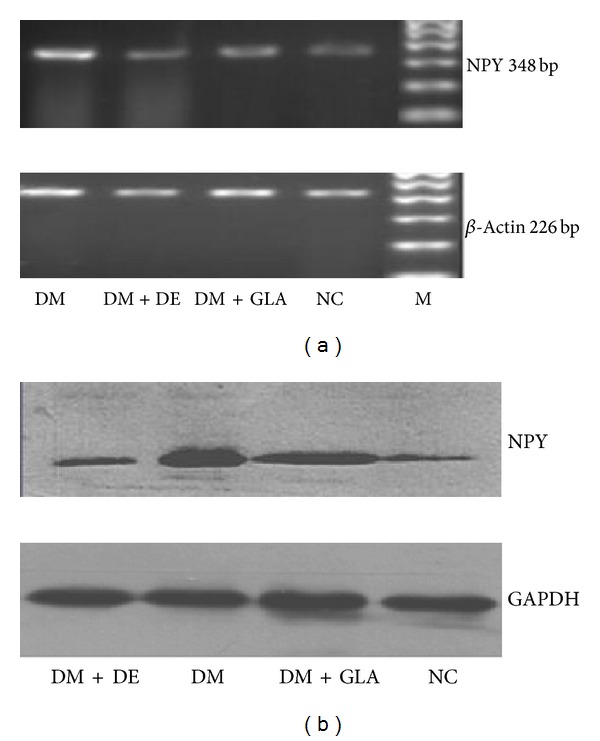
(a) PCR electrophoretogram of NPY mRNA and *β*-actin, (b) the expression of NPY protein and GAPDH. (Note: DM for Diabetes group, DM + DE for detemir-treated diabetes group, DM + GLA for glargine-treated diabetes group, NC for normal control group, and M for marker).

The expression of hypothalamic neuropeptide Y (NPY) mRNA (Figure 1(a)) and protein (Figure 1(b)) in the rats was assessed by RT-PCR and Western blot, respectively. NPY expression in the DM group was increased when compared to the NC group (*P* < 0.01). Following insulin therapy, the NPY levels decreased (*P* < 0.05) and the expression of NPY mRNA and protein in the hypothalamus was lower in the DM + DE group when compared to the DM + GLA group (*P* < 0.05) (Table 4).

### 4.4. The Influence of Insulin on Hypothalamic GAL Expression

**Figure 2:**
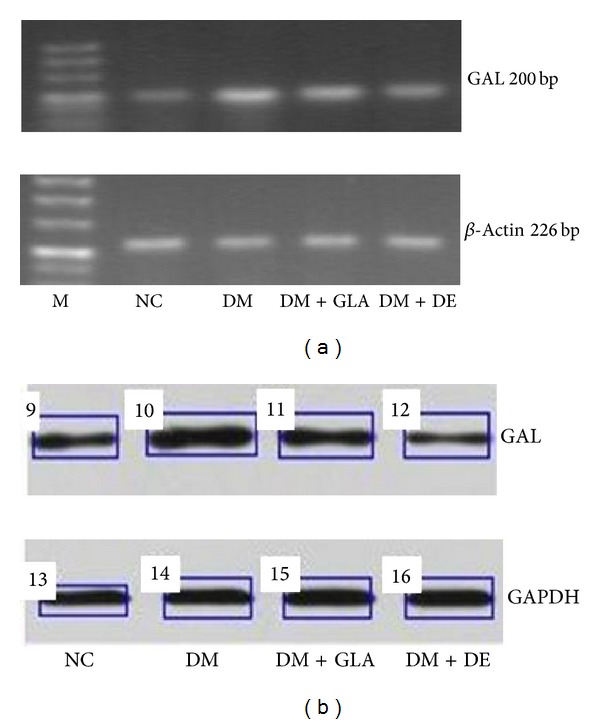
(a) PCR electrophoretogram of GAL mRNA and *β*-actin, (b) the expression of GAL protein and GAPDH. (Note: DM for Diabetes group, DM + DE for detemir-treated diabetes group, DM + GLA for glargine-treated diabetes group, NC for normal control group, and M for marker).

The expression of hypothalamic galanin (GAL) mRNA (Figure 2(a)) and protein (Figure 2(b)) in the DM group was increased compared to the NC group (*P* < 0.01). Following insulin therapy, the GAL levels decreased (*P* < 0.05) and expression of hypothalamic GAL mRNA and protein was lower in the DM + DE group when compared to the DM + GLA group (*P* < 0.05) (Table 5).

## 5. Discussion

To our knowledge, this study is the first to explore the molecular mechanisms regulating lower weight gain in diabetics treated with insulin detemir when compared to those treated with insulin glargine. Results presented here indicate that glycemic control resulting from basal insulin is consistent with previous studies. Weight gain is one of the major problems associated with insulin therapy. This adverse effect raises reluctance in patients to begin insulin treatment and oftentimes treatment will not be initiated. Results from this study demonstrate that insulin detemir and insulin glargine caused altered feeding behaviors in a type 2 diabetes rat model. Following 4 weeks of treatment, the rats in the diabetes plus detemir (DM + DE) group had significantly reduced food intake when compared to the diabetes plus galanin (DM + GLA) group (difference of 4.2 ± 0.3 g/day/rat). Interestingly, when body weight was compared between the two groups, the results indicated that the DM + DE group also gained weight in contrast to the normal control (NC) group but gained less weight than the DM + GLA group. The difference in weight gain between the DM + DE and DM + GLA groups was 36.5 ± 10.6 g. This weight gain difference complements the feeding patterns observed for both insulin intervention groups. Therefore, these results suggest that the level of anorexia induced by insulin detemir is higher than that induced by insulin glargine. A 52-week, open-label, parallel-group, noninferiority, treat-to-target trial by Hollander et al. revealed that mean weight gain was significantly lower with insulin detemir when compared to insulin glargine (2.8 versus 3.8 kg; mean difference, −1.04; 95% CI, −2.08 to −0.01; *P* < 0.05) [20].

In order to elucidate the molecular mechanisms regulating the satiety effect of detemir, neuropeptide Y (NPY) and galanin (GAL), two hypothalamic neuropeptides known to regulate appetite were studied since it was previously established that insulin inhibits synthesis of NPY [21]. Results shown here demonstrate increased expression of NPY mRNA and protein in the diabetes control (DM) group in contrast to the NC group (*P* < 0.01) which corresponds with the major diabetic symptom of overeating. Results from this study also show that NPY expression was lower in the DM + DE group when compared to the DM + GLA group (*P* < 0.05). These findings may partially account for less food intake by the rats in the detemir intervention group. Sindelar et al. investigated the contribution of NPY to pathogenesis of diabetic hyperphagia. The investigators compared the food intake patterns of NPY deficient and wild type mice after inducing diabetes with streptozotocin (STZ). The study showed that after 2 weeks of diabetes onset, the food intake of wild mice was increased by 50% when compared to NPY deficient mice [22]. These results demonstrate that NPY is a potent orexigen and an important physiological food intake regulator.

Galanin (GAL) is also an appetite regulating neuropeptide largely localized in the paraventricular nucleus of the hypothalamus. An injection of GAL into the lateral ventricle or the paraventricular nucleus is reported to increase fat consumption; however, this condition can be reversed by a ventricular injection of insulin [23, 24]. Therefore, we also investigated GAL expression in each treatment group in this study. The results demonstrated that expression of GAL in each study group corresponded with NPY expression. The expression of GAL in the DM group was higher than in the NC group (*P* < 0.01), and expression of GAL in the insulin detemir intervention group was lower when compared to the glargine intervention group (*P* < 0.05). Taken together, decreased expression of NPY and GAL in the hypothalamus after detemir treatment is associated with less food intake in type 2 diabetic mice. We hypothesize that decreased hypothalamic NPY and GAL is a driver for the mechanism of less weight gain following treatment with insulin detemir.

There are hypotheses that may explain the effect of insulin detemir on the expression of NPY and GAL in the hypothalamus. Hollander Pa reported that insulin detemir has faster transport across the blood brain barrier (BBB) due to the fatty acid chain in its structure [25]. Moreover, the lipophilic fatty acid side chain of insulin detemir as well as low albumin content of CSF allows increased access to CNS receptors, which suggests increased CNS penetration by insulin detemir [26]. Access to CNS receptors provides better opportunity for insulin detemir to act centrally on hypothalamic NPY and GAL. This may explain our findings that treatment with detemir resulted in lower expression of NPY and GAL in type 2 diabetic mice when compared to those treated with glargine.

A study investigated the changes in NPY and GAL peptides at simulated high altitude (HA) and their possible role in anorexia in male SD rats. In this study, NPY and GAL levels were estimated in different parts of the brain and plasma of exposed and unexposed control animals. The results indicated that exposed animals had lower levels of these peptides in the hypothalamus and plasma and a significant reduction in food intake. The study concluded that altered expression of these peptides might promote anorexia at HA [27]. Additional research has shown that insulin reduced the expression of both GAL and NPY and insulin applied to medial hypothalamic fragments in vitro significantly reduced GAL and NPY release [24].

This study and others have demonstrated a relationship between feeding behavior and insulin. This is the first report of the impact of insulin detemir and insulin glargine on hypothalamic NPY and GAL. Decreased expression of NPY and GAL corresponded with altered feeding behaviors and weight changes in a type 2 diabetes rat model. Insulin detemir was comparable with insulin glargine in achieving an optimal glycemic level, but insulin detemir reduced weight gain in the animals. The results presented here demonstrate that less weight gain caused by insulin detemir corresponded to downregulation of hypothalamic NPY and GAL, although the mechanism of action is not clear. Further exploration of the molecular mechanisms regulating the effects of insulin detemir on weight gain in diabetic patients, particularly for those who are overweight or obese, will provide evidence for clinical applications of insulin detemir.

## Figures and Tables

**Table 1:** Changes in food intake (g/day/rat) during insulin treatment ($\bar{x}\pm s$).

Groups	At start food intake	1st week food intake	2nd week food intake	4th week food intake
NC group	21.2 ± 1.1	22.2 ± 0.7	24.3 ± 0.5	27.5 ± 1.0∗
DM group	36.7 ± 0.8	41.8 ± 2.8	52.2 ± 2.2	50.5 ± 1.7
DM + DE group	36.1 ± 2.8	40.9 ± 4.2	40.2 ± 4.2	34.4 ± 1.8∗
DM + GLA group	37.1 ± 1.9	41.1 ± 2.6	42.4 ± 3.1	38.6 ± 2.1^∗#^

Note: compared with DM group, ∗means *P* < 0.01; compared with DM + DE group, ^#^means *P* < 0.05.

**Table 2:** Weight change of rats (g) after insulin treatment ($\bar{x}\pm s$).

	NC group weight	DM group weight	DM + DE group weight	DM + GLA group weight
Original model	468.0 ± 15.6	341.3 ± 33.8	341.7 ± 31.4	340.7 ± 29.3
After 4 weeks of insulin treatment	512.3 ± 21.4∗	306.5 ± 22.5	345.0 ± 15.8∗∗	381.5 ± 26.4^∗#^

Note: compared with DM group, ∗means *P* < 0.001 and ∗∗means *P* < 0.05; compared with DM + DE group, ^#^means *P* < 0.05.

**Table 3:** Glycemic level (mmol/L) after 4 weeks of insulin intervention ($\bar{x}\pm s$).

Groups	Glycemic level after 4 weeks of insulin intervention
NC group	4.7 ± 0.7∗
DM group	23.4 ± 1.9
DM + DE group	15.6 ± 1.7∗
DM + GLA group	15.9 ± 1.3∗

Note: compared with DM group, ∗means *P* < 0.01; no significant difference between DM + DE group and DM + GLA group (*P* > 0.05).

**Table 4:** The relative amount of NPY mRNA and protein ($\bar{x}\pm s$).

	NC group	DM group	DM + DE group	DM + GLA group
NPY mRNA/*β*-actin	0.4 ± 0.3^△^	1.8 ± 0.5	0.8 ± 0.5^△^	1.3 ± 1.0^▲▪^
NPY protein/GAPDH	0.6 ± 0.2^△^	1.6 ± 0.2	0.8 ± 0.1^△^	1.0 ± 0.2^△▪^

Note: compared with DM group, ^△^means *P* < 0.01 and ^▲^means *P* < 0.05; compared with DE group, ^▪^means *P* < 0.05.

**Table 5:** The relative amount of GAL mRNA and protein ($\bar{x}\pm s$).

	NC group	DM group	DM + GLA group	DM + DE group
GAL mRNA/*β*-actin	1.32 ± 0.16∗∗	2.00 ± 0.18	1.64 ± 0.15^∗#^	1.13 ± 0.38∗
GAL protein/GAPDH	0.40 ± 0.17∗∗	1.14 ± 0.20	0.74 ± 0.35^∗#^	0.35 ± 0.20∗

Note: compared with DM group, ∗∗means *P* < 0.01 and ∗means *P* < 0.05; compared with DM + DE group, ^#^means *P* < 0.05.

## Abbreviations

T2DM: Type 2 diabetes mellitus
NC: Normal control group
DM: Diabetic control group
DM + DE: Diabetes plus detemir group
DM + GLA: Diabetes plus glargine group
STZ: Streptozotocin
NPY: Neuropeptide Y
GAL: galanin
ICV: Intracerebroventricular
HFD: High-fat diet.
